# A vaccine strategy with multiple prostatic acid phosphatase-fused cytokines for prostate cancer treatment

**DOI:** 10.3892/or.2015.3770

**Published:** 2015-01-29

**Authors:** KEI FUJIO, MASAMI WATANABE, HIDEO UEKI, SHUN-AI LI, RIE KINOSHITA, KAZUHIKO OCHIAI, JUNICHIRO FUTAMI, TOYOHIKO WATANABE, YASUTOMO NASU, HIROMI KUMON

**Affiliations:** 1 Department of Urology, Okayama University, Okayama, Japan; 2 Center for Innovative Clinical Medicine, Okayama University Hospital, Okayama University, Okayama, Japan; 3 Department of Biotechnology, Graduate School of Natural Science and Technology, Okayama University, Okayama, Japan; 4 Department of Veterinary Nursing and Technology, School of Veterinary Science, Nippon Veterinary and Life Science University, Musashino-shi, Tokyo, Japan

**Keywords:** prostate cancer, vaccine, prostatic acid phosphatase, cytokine, fusion protein

## Abstract

Immunotherapy is one of the attractive treatment strategies for advanced prostate cancer. The US Food and Drug Administration (FDA) previously approved the therapeutic vaccine, sipuleucel-T, which is composed of autologous antigen-presenting cells cultured with a fusion protein [prostatic acid phosphatase (PAP) and granulocyte-macrophage colony-stimulating factor (GMCSF)]. Although sipuleucel-T has been shown to prolong the median survival of patients for 4.1 months, more robust therapeutic effects may be expected by modifying the vaccination protocol. In the present study, we aimed to develop and validate a novel vaccination strategy using multiple PAP-fused cytokines for prostate cancer treatment. Using a super gene expression (SGE) system that we previously established to amplify the production of a recombinant protein, significant amounts of PAP-fused cytokines [human GMCSF, interleukin-2 (IL2), IL4, IL7 and mouse GMCSF and IL4] were obtained. We examined the activity of the fusion proteins *in vitro* to validate their cytokine functions. A significant upregulation of dendritic cell differentiation from monocytes was achieved by PAP-GMCSF when used with the other PAP-fused cytokines. The PAP-fused human IL2 significantly increased the proliferation of lymphocytes, as determined by flow cytometry. We also investigated the *in vivo* therapeutic effects of multiple PAP-fused cytokines in a mouse prostate cancer model bearing prostate-specific antigen (PSA)- and PAP-expressing tumors. The simultaneous intraperitoneal administration of PAP-GMCSF, -IL2, -IL4 and -IL7 significantly prevented tumor induction and inhibited the tumor growth in the PAP-expressing tumors, yet not in the PSA-expressing tumors. The *in vivo* therapeutic effects with the multiple PAP-fused cytokines were superior to the effects of PAP-GMCSF alone. We thus demonstrated the advantages of the combined use of multiple PAP-fused cytokines including PAP-GMCSF, and propose a promising prostatic antigen-vaccination strategy to enhance the therapeutic effects.

## Introduction

Prostate cancer is a commonly diagnosed malignant disease in males worldwide, and there are no effective treatment options once the cancer becomes metastatic and refractory to hormonal therapy and chemotherapy. In prostatic tissues, including both the normal prostate and prostate cancer, the expression of several specific proteins, such as prostate-specific antigen (PSA), prostate-specific membrane antigen (PSMA), prostatic acid phosphatase (PAP) and prostate secretory protein-94 (PSP94) has been described (1–3). By targeting these prostate-specific proteins to establish anti-tumor vaccines, immunotherapy has been developed, and has recently been suggested to have potential for the treatment of prostate cancer (4–7).

PAP is a secretory prostate-specific protein that consists of 354 amino acids with an estimated molecular mass of 41 kDa (1,2). Homologs of the human PAP gene have been identified in rats and mice, and these proteins respectively share 75 and 81% homology with the human protein at the amino acid level (2). PAP is overexpressed in 95% of prostate cancer tissues (8), and the tissue specificity of PAP makes it an attractive target antigen for immunotherapy against prostatic malignancy (8,9). Several vaccine candidates have been developed based on the PAP protein using cell-based medicine (10,11), DNA vaccines (12) or peptide antigens (13,14).

Using the full-length PAP protein as a vaccine target, sipuleucel-T (Provenge^®^; Dendreon Inc., Seattle, WA, USA), an autologous active cellular immunotherapy, was developed for the treatment of metastatic castration-resistant prostate cancer (10). The cellular agent is manufactured from individual peripheral monocytes enriched by leukapheresis, and is an antigen-presenting cell vaccine loaded *ex vivo* with a fusion protein linking PAP to granulocyte-macrophage colony-stimulating factor (GMCSF) (15,16). After incubation with the PAP-GMCSF fusion protein, the immunologically activated cells are intravenously administered to patients every two weeks for a total of three infusions. A placebo-controlled randomized phase III trial demonstrated that the personalized cell-based vaccine showed evidence of efficacy in reducing the risk of death among patients with prostate cancer refractory to hormonal therapy (10). In the patients treated with sipuleucel-T, there was a relative reduction of 22% in the risk of death compared with the placebo group, representing a 4.1-month improvement in median survival (25.8 months in the sipuleucel-T group vs. 21.7 months in the placebo group). Based on the results of a phase III study, the US Food and Drug Administration (FDA) approved sipuleucel-T for the treatment of prostate cancer in April 2010, and this was the first antigen-specific immunotherapy officially approved in a developed country.

Although sipuleucel-T can prolong the overall survival in patients with progressive prostate cancer, more robust immunological effects seem to be possible that can further improve survival. In order to enhance the therapeutic effects of the PAP-GMCSF fusion protein, we experimentally tried to modify the methods and to develop a novel vaccination strategy with the additional use of multiple PAP-fused cytokines, including human interleukin-2 (IL2), IL4 and IL7. The reason for adding these interleukins is that these cytokines can potently activate anticancer immune cells and upregulate the effects of the therapeutic vaccination for cancer treatment (17–19).

The cost for sipuleucel-T is reported to be 93,000 US$ per course of treatment, thus making this therapeutic vaccine an expensive treatment option (11). One of the ways to reduce the pharmacological cost is to increase the production efficiency of the PAP-GMCSF fusion protein. Since we previously established a super gene expression (SGE) cassette to amplify the production of a recombinant protein in 293-F cells (20,21), we herein attempted to apply this mammalian expression system to produce multiple PAP-fused cytokines. In the present study, we validated the activity of the PAP-fused proteins as cytokines and demonstrated the advantages of the combined use of multiple PAP-fused cytokines in *in vitro* and *in vivo* situations.

## Materials and methods

### Construction of the SGE plasmid vectors encoding PAP-fused cytokines

**Figure 1:**
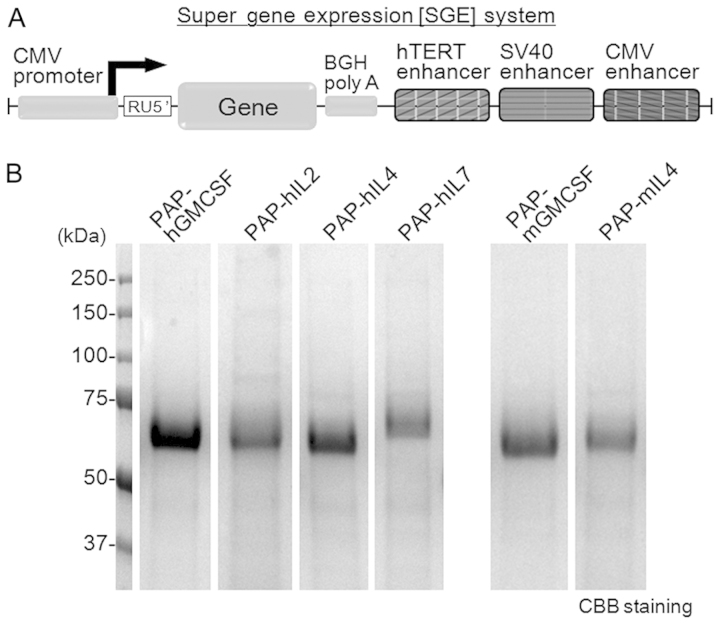
(A) A schematic diagram of the super gene expression (SGE) system. In this SGE system, triple translational enhancer sequences of hTERT, SV40 and the CMV enhancer were inserted downstream of the BGH polyA sequence. (B) PAP-fused human (h) or mouse (m) cytokines were produced by the SGE system in the 293-F cells with serum-free culture medium. To examine the amount and purity of the recombinant PAP-fused cytokines in the medium, the supernatant on day 5 after transfection was analyzed by SDS-PAGE with Coomassie brilliant blue (CBB) staining. BGH polyA, bovine growth hormone polyadenine nucleotides.

The construction of a plasmid vector with the SGE system was performed as previously described (20,21). The gene expression cassette with the CMV promoter, sequences of RU5′, bovine growth hormone polyadenine nucleotides (BGH polyA) and tandem elements of triple translational enhancers hTERT, SV40 and CMV was artificially synthesized and cloned into the pIDT-SMART vector (Integrated DNA Technologies, Inc., Coralville, IA, USA) (Fig. 1A). The RU5′ sequence [269 bp, accession no. J02029 (374–642)] was derived from the R segment and part of the U5 sequence of the HTLV type 1 long terminal repeat and was used to enhance the stability of DNA and RNA and increase the translation efficiency (21). The tandem element of the triple translational enhancers consists of the hTERT enhancer [189 bp, accession no. DQ264729 (1618–1806)], SV40 enhancer [319 bp, accession no. AY864928 (2156–2474)] and CMV enhancer [479 bp, accession no. AJ318513 (159–637)]. The tandem sequence of the hTERT, SV40 and CMV enhancers is the main element of the SGE system. The SGE plasmid encoding a fusion protein of full-length human PAP and one of the cytokines (human GMCSF, IL2, IL4, IL7 or mouse GMCSF or IL4) were then constructed and used to express the recombinant PAP-fused cytokines. The fusion cytokines were designed to be histidine-tagged for purification.

### Preparation of the recombinant PAP-fused cytokines

The recombinant PAP-fused cytokines were expressed by the FreeStyle 293 expression system (Invitrogen, Carlsbad, CA, USA) with the respective SGE plasmids according to the manufacturer’s instructions. The expression system was designed to allow the transfection of suspended 293-F cells (derived from HEK293 cells) in serum-free culture medium. Transient transfection with the plasmid vector was performed, and the secreted recombinant fusion protein in the culture supernatant was collected. The PAP-fused cytokines were purified using the histidine tag included in the fusion protein, as previously described (22). The recombinant PAP-fused cytokines were stocked and maintained at −80°C until use. The PAP-fused human and mouse cytokines were analyzed for the protein amount, purity and concentration by examining the densitometry of the bands on SDS-PAGE with Coomassie brilliant blue (CBB) staining.

### Preparation and culture conditions of blood mononuclear cells

Human peripheral blood mononuclear cells (PBMCs) were prepared from the blood of healthy donors by the standard procedure using Ficoll-Paque centrifugation (22). This research was carried out on humans following the international and national regulations. Written informed consent was obtained from the subjects. For some experiments, human CD14-positive monocytes were purchased from Lonza (Walkersville, MD, USA). Mouse blood was obtained from the inferior vena cava, and the mononuclear cells were prepared by Ficoll-Paque centrifugation. The blood mononuclear cells and CD14-positive monocytes were cultured either in LGM-3 medium (Lonza) alone or in the presence of recombinant PAP-fused cytokines under the indicated conditions. As a positive control for the differentiation of human dendritic cells, the PBMCs and CD14-positive monocytes were cultured in LGM-3 medium supplemented with human granulocyte-macrophage colony-stimulating factor (hGMCSF; 2 ng/ml) and human interleukin-4 (hIL4; 2 ng/ml) (both from R&D Systems, Minneapolis, MN, USA) (17,22). The cells were cultured at 37°C in humidified incubators containing air with 5% CO_2_.

### Proliferation assay using TF-1 cells

TF-1, a human GMCSF-dependent proliferative cell line (23,24), was used to analyze the cytokine activity of PAP-hGMCSF. TF-1 cells (10^4^ cells/well) were plated on 96-well plates and cultured as previously reported (23). The PAP-hGMCSF was added to the culture medium at the indicated concentrations derived from a 3-fold serial dilution. hGMCSF was used as a positive control for the GMCSF-dependent proliferation. After three days of incubation, the proliferation of the TF-1 cells was analyzed with the 3-(4,5-dimethylthiazol-2-yl)-2,5-diphenyltetrazolium bromide (MTT) assay, according to the manufacturer’s instructions.

### Flow cytometric analysis

To analyze the proliferation of CD8^+^, CD4^+^ and CD19^+^ lymphocytes, flow cytometry was performed as previously described (22). Human PBMCs (75×10^4^ cells/well) were incubated with PAP-hIL2 (1 μg/ml) for four days on 6-well plates, and then the floating cells were examined for each surface antigen. The cells were stained with the following FITC-conjugated antibodies for 60 min on ice: CD8 (551347; BD Pharmingen, San Diego, CA, USA) as a marker for cytotoxic T lymphocytes, CD4 (555346) for helper T lymphocytes and CD19 (555412) for B lymphocytes. After staining, 2×10^4^ cells were acquired on a FACSCalibur flow cytometer and analyzed using the CellQuest software program (both from Becton-Dickinson, Franklin Lakes, NJ, USA).

### Animal experiments

The RM9 mouse prostate cancer cell line was kindly provided by Dr T.C. Thompson (The University of Texas, Houston, TX, USA) (25) and was used in the animal experiments. In our experimental set-up, we prepared PSA-RM9 and PAP-RM9 cells stably expressing the human PSA or PAP protein, respectively. The stable clones of RM9 cells were established by the transfection of plasmids encoding the full-length human PSA or PAP genes and a neomycin-resistance gene, as previously described (20,21,26). Using the PSA-RM9 and PAP-RM9 cells, we developed a mouse prostate cancer model bearing both PSA- and PAP-expressing tumors. The *in vivo* experimental schedule and treatments with the PAP-fused cytokines are shown in Fig. 4A. Since IL2 and IL7 possess cross-reactivity between human and mouse species (27–29), we used PAP-human IL2 and PAP-human IL7 instead of PAP-mouse IL2 and PAP-mouse IL7, respectively. A total of nine intraperitoneal administrations of the PAP-fused cytokines were performed. On day 7, the PSA-RM9 and PAP-RM9 cells were subcutaneously inoculated into the left and right femurs, respectively, of C57BL/6 adult male mice. All mice were examined for the tumor formation and for the tumor size on day 21, and then were sacrificed. Animal experiments using anticancer cytokines were approved by the Animal Care and Use Committee, Okayama University.

### Statistical analysis

The data are expressed as the means ± standard error. Student’s t-test or the Chi-square test was used to determine the statistical significance of differences between the two groups. Differences were considered to be statistically significant for values of p<0.05.

## Results

### Production of recombinant multiple PAP-fused cytokines using the SGE system

We previously developed the SGE system in order to improve the protein production by conventional gene expression systems (20,21). In the gene expression cassette with the SGE system, triple translational enhancer sequences of hTERT, SV40 and the CMV enhancer were inserted downstream of the BGH polyA sequence (Fig. 1A). We recently reported that the SGE system significantly enhanced adenoviral vector-mediated gene expression (~2- to 5-fold) in human cancer cell lines, as determined by western blot analysis) in comparison to the system using conventional gene expression (20). Therefore, we herein attempted to apply this mammalian expression system to produce the multiple PAP-fused cytokines.

The amount of protein produced and the purity of the recombinant PAP-fused cytokines produced by the SGE system were analyzed by SDS-PAGE with CBB staining (Fig. 1B). As a result, a significant amount of PAP-fused cytokines was obtained in the serum-free culture medium of the 293-F cells on day 5 by the transient gene expression. The recombinant fusion proteins were recognized as single bands by gel electrophoresis. The concentration (mg/l) of the fusion proteins in the medium was calculated: PAP-hGMCSF, 123.3; PAP-hIL2, 91.7; PAP-hIL4, 96.9; PAP-hIL7, 64.5; PAP-mGMCSF, 94.2; PAP-mIL4, 62.8.

### Recombinant PAP-fused cytokines maintain their cytokine activity

**Figure 2:**
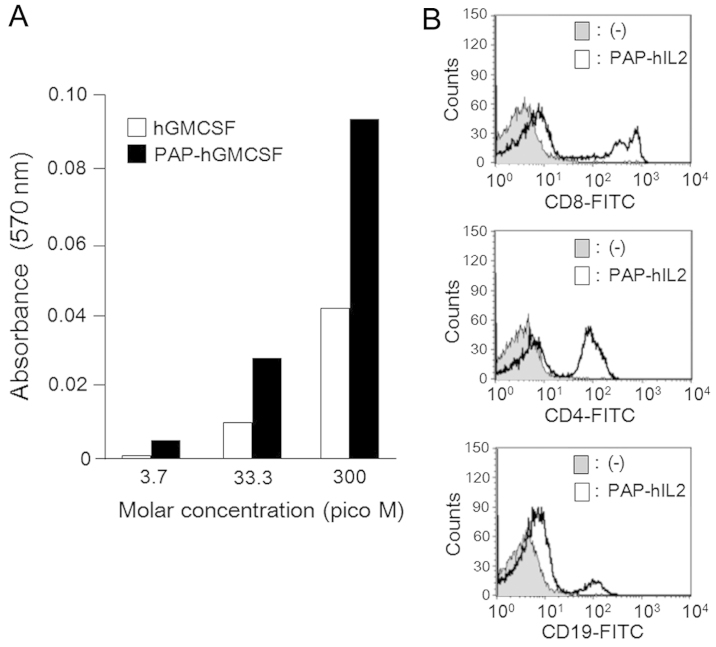
(A) Growth dependency of TF-1 cells on human (h)GMCSF and PAP-hGMCSF was analyzed. The cell proliferation at the indicated concentrations was assayed by determining the absorbance at 570 nm in the MTT assay. (B) Proliferation of CD8^+^, CD4^+^ and CD19^+^ lymphocytes following treatment with PAP-hIL2 was analyzed by flow cytometry. Human peripheral blood mononuclear cells (PBMCs) were incubated with PAP-hIL2 (1 μg/ml) for four days, and then the floating cells were examined for each surface antigen. GMCSF, granulocyte-macrophage colony-stimulating factor.

In order to confirm the cytokine activity of the recombinant PAP-fused cytokines, we conducted *in vitro* experiments with PAP-hGMCSF and PAP-hIL2. It is known that human GMCSF stimulates the proliferation of TF-1 cells (23,24). Therefore, we first analyzed the growth dependency of TF-1 cells by adding PAP-hGMCSF in the culture medium. The cell proliferation at the indicated concentrations was assessed by determining the absorbance of the cells in the MTT assay. The results indicated that proliferation of the TF-1 cells was increased in a dose-dependent manner by PAP-hGMCSF (Fig. 2A). We next investigated the IL2 activity of PAP-hIL2 by evaluating the proliferation of lymphocyte lineages. The cultivation of human PBMCs in the presence of PAP-hIL2 resulted in the rapid growth of the CD8^+^, CD4^+^ and CD19^+^ lymphocytes within four days, as determined by flow cytometry (Fig. 2B). These results indicate that the recombinant PAP-fused cytokines produced by the SGE system retain the usual cytokine activity.

### Differentiation of dendritic cells is enhanced by the combined use of multiple PAP-fused cytokines, including PAP-GMCSF

**Figure 3:**
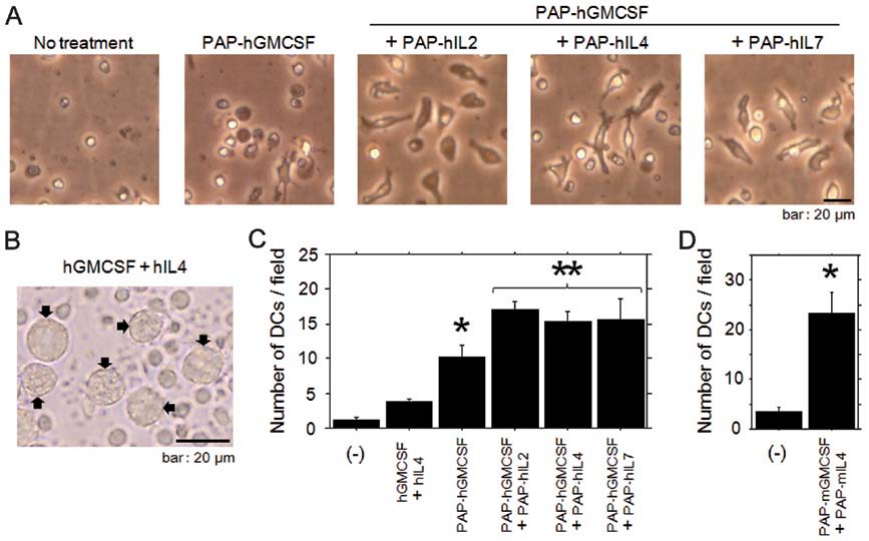
(A) Appearance of human CD14-positive monocytes developing into dendritic cells was evaluated. For the treatments, each PAP-fused human cytokine was added at a concentration of 1 μg/ml. The morphology of the cells on day 1 was photographed under a phase contrast microscope. (B) The appearance of human dendritic cells (arrow) differentiated from peripheral blood mononuclear cells (PBMCs) is shown. The dendritic cells were induced by the incubation with hGMCSF and hIL4 (each, 2 ng/ml) for seven days. The morphology of the cells on day 7 was photographed under a phase contrast microscope. (C) Differentiation of human dendritic cells was quantified for the different treatment groups. The monocytes were treated with hGMCSF and hIL4 (each, 2 ng/ml) or with the PAP-fused cytokines (each, 1 μg/ml) for three days. The number of cells developing into dendritic cells was counted for five randomly selected fields under a microscope, and the results are shown. ^*^Significant difference was observed in comparison to the treatment with hGMCSF and hIL4. ^**^Significant difference was observed in comparison to the treatment with PAP-hGMCSF alone. (D) Differentiation of mouse dendritic cells was quantified for the different treatment groups. The blood mononuclear cells were treated with PAP-fused mouse (m) cytokines (each, 5 μg/ml) for seven days. The number of cells developing into dendritic cells was counted for five randomly selected fields under a microscope, and the results are shown. ^*^Significant difference was observed in comparison to the mononuclear cells treated with medium alone. hGMCSF, human granulocyte-macrophage colony-stimulating factor.

To confirm the ability of the multiple PAP-fused cytokines to induce the differentiation of monocytes into dendritic cells, purified monocytes from PBMCs were cultured in the presence of recombinant fusion cytokines. Since GMCSF has been considered to be an essential cytokine for the differentiation and maturation of dendritic cells (17), we basically used PAP-GMCSF and attempted to combine the other PAP-fused cytokines with it in order to augment the immunological effects. During the first two days of treatment with the multiple PAP-fused cytokines (including PAP-hGMCSF), most of the monocytes displayed cellular elongation (Fig. 3A). The ratio of elongated monocytes in samples treated with the multiple fusion cytokines was higher than that in the cells treated with PAP-hGMCSF alone. With regard to the monocytes cultivated with medium alone, there were only a few cells with dendritic-cell like features.

We next quantified the dendritic cell differentiation induced by the treatment with multiple PAP-fused cytokines, including PAP-GMCSF. Since it has been well established that dendritic cells can be differentiated by incubation with GMCSF and IL-4 (17,22), we referred to the morphological features of cells treated with these cytokines as a positive control for dendritic cells (Fig. 3B). After the incubation with PAP-fused human cytokines, the number of cells developing into human dendritic cells was counted in several microscopic fields. When treated with the multiple fused cytokines, the number of developing dendritic cells was significantly increased in comparison to those treated with PAP-GMCSF alone (Fig. 3C). The enhanced differentiation of mouse dendritic cells was also confirmed by cultivating the blood mononuclear cells with multiple fusion cytokines, including PAP-mGMCSF and PAP-mIL4 (Fig. 3D). These results indicated that the differentiation of dendritic cells was enhanced by the combined use of multiple PAP-fused cytokines, including PAP-GMCSF.

### In vivo therapeutic effects are enhanced by the combined use of multiple PAP-fused cytokines, including PAP-GMCSF

**Figure 4:**
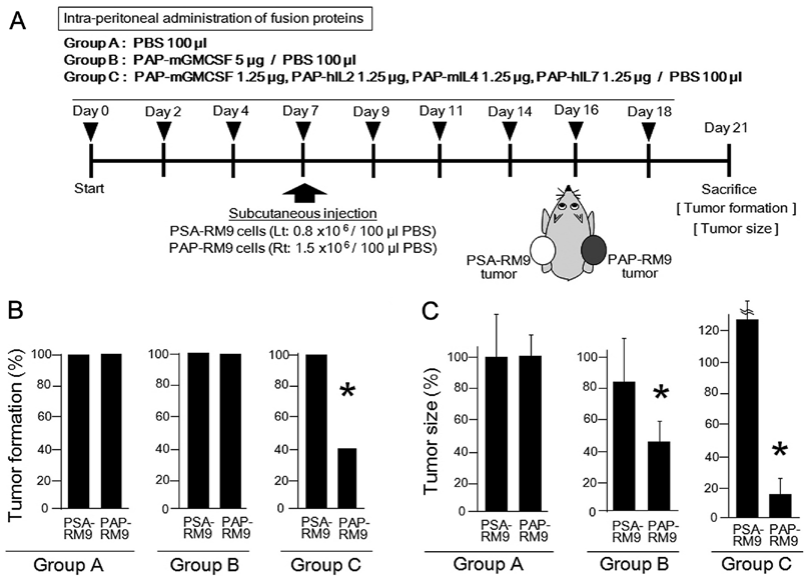
(A) *In vivo* experimental schedule and treatments with PAP-fused cytokines are shown. (A–C) Treatments were performed intraperitoneally and the animals were separated into three groups. On day 7, mouse prostate cancer RM9 cells stably expressing human PSA and PAP (PSA-RM9 and PAP-RM9 cells) were subcutaneously inoculated into the left and right femurs of C57BL/6 adult male mice, respectively. The PSA-expressing tumors derived from PSA-RM9 cells were monitored as a negative control for the PAP-based immunological treatments. All mice were examined for tumor formation and tumor size on day 21. PBS, phosphate-buffered saline. (B) Antitumor effects of the treatment are shown with regard to preventing tumor formation. The incidence of PSA-RM9 and PAP-RM9 tumor formation was analyzed on day 21. ^*^Significant difference was observed in comparison to the tumor formation (%) of the PAP-RM9 in group A. (A–C) Each treatment group comprised five mice. (C) Antitumor effects of the treatments are shown with regard to the suppression of tumor growth. The PSA-RM9 and PAP-RM9 tumor sizes were analyzed on day 21. The values are represented as the percentages of the respective PSA-RM9 or PAP-RM9 tumor sizes in group A. ^*^Significant difference was observed in comparison to the tumor size of the PAP-RM9 tumors in group A. PSA, prostate-specific antigen; PAP, prostatic acid phosphatase.

The *in vitro* results in the present study indicated the possibility that a PAP-fused cytokine-based *in vivo* increase in immune cells may upregulate the anticancer immune response by targeting the PAP molecule. In order to investigate the antitumor effects of PAP-fused cytokines, *in vivo* experiments were performed using a mouse prostate cancer model bearing both PSA- and PAP-expressing tumors (Fig. 4A). As a PAP-targeting vaccine, the multiple PAP-fused cytokine treatment would be expected to be most effective for use against the initial stages of prostate cancer or for minimal disease. Therefore, the vaccine was started to be administered before the cancer cell inoculation in our preclinical mouse model.

In the preliminary experiments, *in vitro* and *in vivo* stable expression of the PSA and PAP proteins was confirmed for the PSA-RM9 and PAP-RM9 cells, respectively (data not shown). Since IL2 and IL7 possess cross-reactivity between humans and mice (27–29), we used PAP-human IL2 and PAP-human IL7 instead of PAP-mouse IL2 and PAP-mouse IL7, respectively. We first analyzed whether the PAP-fused cytokines could protect mice against challenge with a PAP-expressing tumor. The incidence of PSA-RM9 and PAP-RM9 tumor formation was analyzed on day 21 after the subcutaneous inoculation of cancer cells. In the present study, PSA-expressing tumors derived from PSA-RM9 cells were monitored for the effects of the PAP-based immunological treatments. The co-administration of PAP-GMCSF, -IL2, -IL4 and -IL7 significantly prevented the tumor induction of PAP-RM9 cancer cells (Fig. 4B). On the other hand, the treatment with PAP-GMCSF alone failed to prevent the tumor formation.

We next investigated whether PAP-fused cytokines inhibits PAP-RM9 tumor growth *in vivo*. Significant inhibition of the tumor growth was observed in the groups treated with both PAP-GMCSF alone and with the co-administration of PAP-GMCSF, -IL2, -IL4 and -IL7 (Fig. 4C). The *in vivo* therapeutic effects of the multiple PAP-fused cytokines were superior to the effects observed with PAP-GMCSF treatment alone. For the control PSA-expressing tumors derived from PSA-RM9 cells, no significant therapeutic effect was observed following the treatments with PAP-fused cytokines. Based on the results of the tumor challenge and growth, it was concluded that PAP-specific immune activation occurred in the mice treated with the PAP-fused cytokines, and the antitumor effects were significantly enhanced by the additional cytokine (IL2, IL4 and IL7) fusion proteins.

## Discussion

In the present study, we experimentally validated a PAP-GMCSF-based vaccine strategy with multiple PAP-fused cytokines for the immunotherapy of prostate cancer. We also demonstrated the availability of the SGE system for the production of recombinant PAP-fused cytokines. With regard to the SGE system, we originally developed it as a gene expression system that allows the gene of interest to be expressed with very high efficiency in 293-F cells (20,21). In this SGE construct of the vectors, the linkage of triple translational enhancer sequences of hTERT, SV40 and CMV enhancers was inserted into a site downstream of the sequence of the BGH polyA. The CMV promoter driving SGE system robustly enhanced the gene expression of plasmid and adenoviral vectors in comparison to the cassette with CMV promoter alone (20). The superiority of the CMV promoter-SGE system was also observed compared to the gene expression cassette with EF-1α and CAG promoters alone (21). Using this SGE system, we succeeded in producing a significant amount of the PAP-fused cytokines (human GMCSF, IL2, IL4, IL7, and mouse GMCSF and IL4) in the medium of 293-F cells. The recombinant proteins were easily concentrated for use in the experiments and demonstrated activity as cytokines.

We herein examined the activity of the fusion proteins as cytokines *in vitro*, and significant upregulation of dendritic cell differentiation from monocytes was achieved by treatment with PAP-GMCSF when it was used with the other PAP-fused cytokines. The PAP-fused human IL2 led to significantly increased proliferation of T and B lymphocytes, as determined by flow cytometry. We thus validated the cytokine functions of the PAP-fused cytokines. Dendritic cells are antigen-presenting cells that play important roles in anticancer immune responses. GMCSF has been considered to be the main cytokine required for the differentiation and maturation of dendritic cells (17). The combined use of GMCSF and IL4 is the most extensively characterized and utilized combination for the *in vitro* differentiation of dendritic cells from peripheral blood monocytes (17,22). In terms of the effects of the different cytokine combinations with GMCSF on the development of dendritic cells, the addition of IL2 and IL7 also plays important roles (17,30–32). Moreover, previous reports indicate that IL2 induces the differentiation and/or maturation toward a dendritic cell phenotype without GMCSF (31), and could enhance the motility of dendritic cells (30).

**Figure 5:**
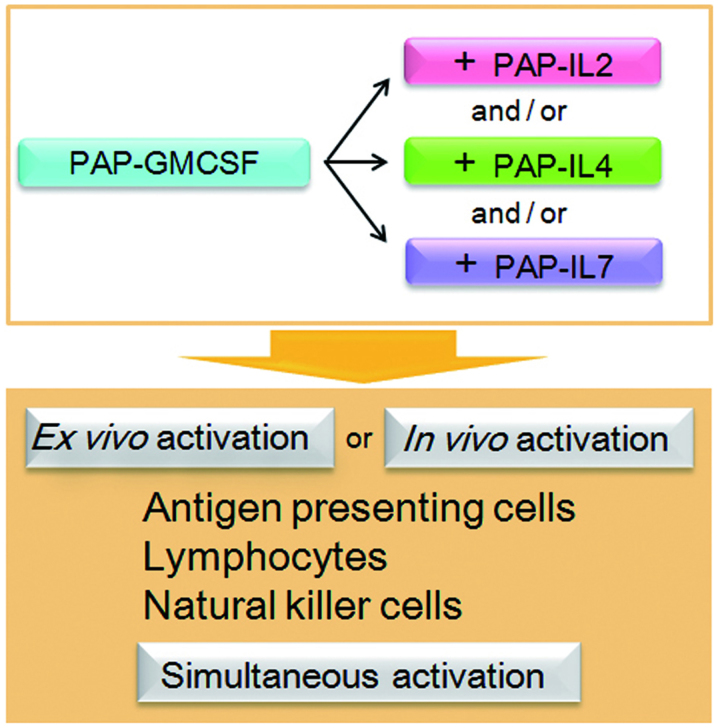
*Ex vivo* and *in vivo* vaccine strategy with multiple PAP-fused cytokines for prostate cancer treatment. The combined use of multiple PAP-fused cytokines, including PAP-GMCSF, activated the anticancer immune cell lineages simultaneously. We propose that the therapeutic effects of the PAP-fused cytokines were enhanced and superior to the effects with PAP-GMCSF alone. PAP, prostatic acid phosphatase; GMCSF, granulocyte-macrophage colony-stimulating factor.

With regard to the other immune cells (other than antigen-presenting cells), CD4^+^ and CD8^+^ T lymphocytes, CD19^+^ B lymphocytes and natural killer (NK) cells all play important roles in antitumor immunity. Interleukins, including IL2, IL4 and IL7, are known to exhibit and enhance antitumor effects, not only through the activation of these antitumor immune cells, yet also via the activation of macrophages and lymphokine-activated killer (LAK) cells (3,33–35). Thus, as a result of the enhanced cancer-specific immunity following the simultaneous activation of these immune cells, the combined use of multiple cytokines is a promising strategy (Fig. 5). Our results also indicate that this strategy could be adaptable for both *ex vivo* activation and *in vivo* activation of the immune cells for immunotherapy. Further studies are warranted toward the clinical application of the PAP-GMCSF-based vaccine strategy with multiple PAP-fused cytokines for prostate cancer treatment.

To further demonstrate the utility of the multiple PAP-fused cytokines, we investigated the *in vivo* therapeutic effects of the recombinant proteins in a mouse prostate cancer model bearing both PSA- and PAP-expressing tumors. The co-administration of PAP-GMCSF, -IL2, -IL4 and -IL7 significantly prevented tumor induction and inhibited tumor growth in the PAP-expressing tumors, yet not in the PSA-expressing tumors. The finding indicates that PAP-specific immune activation occurred in the mice treated with the PAP-fused cytokines. We also demonstrated that the tumor growth suppression by the multiple PAP-fused cytokines was superior to the effect with PAP-GMCSF alone. Therefore, the vaccination against PAP was significantly upregulated by the additional cytokine (IL2, IL4 and IL7) fusion proteins. The *in vivo* results are consistent with our *in vitro* findings showing the increased induction of dendritic cells and lymphocytes by the fusion cytokines. It is conceivable that the PAP-fused cytokines, including PAP-GMCSF, induced the robust differentiation of the monocytes into antigen-presenting dendritic cells, resulting in the activation of PAP-specific CD4^+^ and CD8^+^ T lymphocytes in the treated mice. These activated T lymphocytes are then thought to work against the PAP-expressing tumor lesions in the mouse model, targeting the antigen and mediating the anti-tumor responses. On the other hand, based on the increased induction of CD19^+^ B lymphocytes by the fusion cytokines *in vitro*, the antitumor effects could also be partially due to the humoral immunity of the PAP-specific antibodies induced in the mouse model.

A question that remains to be answered is associated with the peptide epitopes of the PAP antigen in the cellular arm of the immune response in the current C57BL/6 mouse model. Notably, other investigators recently reported that three human PAP epitopes (114–128, 299–313 and 230–244) were immunologically processed for vaccinations in mice (8). They demonstrated that the PAP (114–128) epitope, which is identical between human and mouse species at the amino acid level, elicits CD4^+^ and CD8^+^ T lymphocyte-specific responses in C57BL/6 mice. Furthermore, when administered to mice bearing prostate cancer, the PAP (114–128) peptide prevented and reduced the growth of tumors in the prophylactic and therapeutic settings. These studies showed that the antitumor effects were associated with the infiltration of CD8^+^ tumor-infiltrating lymphocytes, and proposed that PAP (114–128) is a highly relevant peptide on which to base vaccines for the treatment of prostate cancer. Therefore, it is conceivable that the PAP epitopes, including the peptide (114–128), played essential roles in the anti-PAP vaccination processes in our mouse model after treatment. Additionally, we herein adopted a vaccination strategy using the full-length PAP protein as an immunogen. The use of entire proteins brings about significant advantages, since it can allow the immune cells to present multiple epitopes, including unknown epitopes associated with different MHC class I molecules, in addition to helper epitopes associated with MHC class II molecules (36).

The previously described therapeutic vaccine, sipuleucel-T, is composed of autologous antigen-presenting cells cultured with a fusion protein of PAP-GMCSF, and its administration prolonged the overall survival among patients with metastatic castration-resistant prostate cancer (10). Sipuleucel-T has opened a new era for the treatment of prostate cancer (11), and immunotherapy has become one of the attractive treatment strategies for various cancers. The immune responses to the immunized PAP antigen were augmented in patients who received sipuleucel-T (10), indicating that strategies that can enhance the effects of vaccination are attractive for the next step in immunotherapy. We herein demonstrated the advantages of the *in vitro* and *in vivo* combined use of multiple PAP-fused cytokines, including PAP-GMCSF, and therefore propose that the combined use of these fusion proteins could be promising for the enhancement of the immunological effects in prostatic antigen-based vaccination therapy. Moreover, if the super gene expression (SGE) system is applied in the Good Manufacturing Practice (GMP) setting, it could be used for the efficient production and cost-reduction of PAP-fused cytokines for clinical use. Further examinations will be required to apply the current strategy employing the PAP-fused cytokines for *ex vivo* and *in vivo* human immunotherapy.

In conclusion, we demonstrated the advantages of the combined use of multiple PAP-fused cytokines, including PAP-GMCSF, under *in vitro* and *in vivo* experimental conditions, and propose that this prostatic antigen-based vaccination strategy should be promising for future clinical development. The current findings provide immunological insight for enhancing the therapeutic effects of cytokine-based immunotherapy. It should also be noted that the approach using multiple antigen-fused cytokines is adaptable to other cancer types by changing the prostatic PAP antigen to a different cancer-specific antigen.
